# Validation and psychometric properties of the Italian Vaccination Attitudes Examination (VAX-I) scale

**DOI:** 10.1007/s12144-022-03209-5

**Published:** 2022-05-27

**Authors:** Francesco Bruno, Valentina Laganà, Rebecca Pistininzi, Francesca Tarantino, Leslie Martin, Rocco Servidio

**Affiliations:** 1 Regional Neurogenetic Centre (CRN), Department of Primary Care, ASP Catanzaro, Viale A. Perugini, 88046 Lamezia Terme, CZ Italy; 2 Association for Neurogenetic Research (ARN), Lamezia Terme, CZ Italy; 3 Academy of Cognitive Behavioral Sciences of Calabria (ASCoC), Lamezia Terme, CZ Italy; 4 Department of Medical and Surgical Sciences, Magna Graecia University of Catanzaro, Catanzaro, Italy; 5 Department of Psychology, La Sierra University, Riverside, CA USA; 6 Department of Cultures, Education and Society, University of Calabria, Via Pietro Bucci, Cubo 18/B – Quarto Piano, 87036 Arcavacata di Rende, Cosenza Italy

**Keywords:** Theory of planned behaviour, Vaccination attitudes, Vaccination intention, Vaccination behaviour, Vaccine uptake, Vaccine hesitancy, COVID-19, Italian validation, The Vaccination Attitudes Examination (VAX) Scale, VAX-I scale

## Abstract

Despite the massive volume of scientific evidence on the benefits of immunisation, vaccine hesitancy is still a global health threat and represents an obstacle to controlling the spread of viruses such as SARS-CoV-2 and its associated COVID-19. Thus, the present study aimed to adapt and validate an Italian version of the Vaccination Attitudes Examination (VAX) scale. The Italian version of the scale (VAX-I), along with validation measures (general health perceptions, perceived sensitivity to medicines, intention to get the flu vaccine, and trust in health authorities) were administered to a sample of 534 Italian participants aged 18 to 87 (*M* = 32.41, *SD* = 15.35). The original version of the VAX scale was translated into Italian using a back-translation method. The parallel and confirmatory factor analyses showed that the scale’s four-factor structure fits the data well, as in the original version. Reliability coefficients indicated that the VAX-I scale showed good internal consistency and measurement invariance results demonstrated that the VAX-I scale is stable across gender. Construct validity was supported by the significant negative correlation with general health perceptions, intentions to get the flu vaccine, and trust in health authorities, and the weak but significant and positive correlation with perceived sensitivity to medicines. Overall, the VAX-I scale appears to be a valid instrument to assess vaccine hesitancy in the Italian context.

**Supplementary Information:**

The online version contains supplementary material available at 10.1007/s12144-022-03209-5.

## Introduction

Attitudes can be defined as “psychological tendency that is expressed by evaluating a particular entity with some degree of favour or disfavour” (Eagly & Chaiken, 1993, p. 1). Attitudes are related to both intentions and behaviours, eloquently described by the Theory of Reasoned Action (TRA; Ajzen & Fishbein, 1980; Fishbein, 1967; Sheppard et al., 1988) and its subsequent iteration, the Theory of Planned Behaviour (TPB; Madden et al., 1992). A comprehensive body of literature (summarised by meta-analytic findings) supports the idea that attitudes play a crucial role in predicting a wide range of behaviours (Glasman & Albarracin, 2006), including health behaviours (Sheeran et al., 2016). In recent years, attitudes have been examined in Italian samples across various fields such as education (Chiesi & Bruno, 2021), smoking cessation strategies among nurses (Maniscalco et al., 2021), and drug use (Di Gennaro et al., 2020) and have demonstrated that, consistent with prior literature, attitudes across these diverse domains predict outcomes and can be influenced by interventions.

With the outbreak of COVID-19 and the development of vaccines to slow its spread, hesitancy among some groups to receive the vaccine has created a barrier to effectively managing this global public health emergency, and researchers have turned attention to examining attitudes related to vaccinations in order to better address the problem (Azlan et al., 2020, Zhong et al., 2020; Shi et al., 2020).

Since the last century, vaccinations have been used effectively to reduce the rate of infections both among vaccinated individuals (Hajj et al., 2015) and through herd immunity (Fine et al., 2011) as part of efforts to eradicate viral diseases (Fisher et al., 2020). However, the new threat posed by COVID-19 and the need for mass vaccinations comes at a time of growing scepticism about vaccinations—reluctance or refusal to vaccinate despite the availability of the vaccine – especially, but not only, in developed countries (Shacham et al., 2021; Trujillo & Motta, 2021; World Health Organization, 2020). Even before the COVID-19 crisis, the World Health Organization (WHO) ranked hesitation about vaccines as one of the top ten global health threats (World Health Organization, 2019). Therefore, vaccine scepticism could undermine COVID-19 immunisation efforts (Ball, 2020; Taylor et al., 2020). This evidence underlines the urgency of investigating attitudes towards vaccination in the general population (Taylor et al., 2020) and, especially, understanding the determinants of vaccine hesitancy.

There are multiple reasons that people may be hesitant to obtain a vaccine—they may mistrust the intentions of the creators or promoters of vaccines, have concerns about the safety of the vaccinations, or doubt that they are necessary or effective (Brown et al., 2010; Garcini et al., 2012; Yaqub et al., 2014). Because COVID-19 vaccines were tested and brought to market quickly, safety concerns have been especially prominent. During public health crises, demand for information increases, which shapes people’s attitudes and understanding (Thelwall & Stuart, 2007; van der Meer, 2018). Failures to effectively communicate reliable information can hamper the efficient mitigation of a social crisis (Liu & Kim, 2011). In the present case, both the delivery speed and the political overlay have created the perception that COVID-19 vaccines may not be safe, which undermined public confidence in them (Limaye et al., 2021).

A recent study conducted in Italy found that participants who had no intention to receive the COVID-19 vaccine reported lower levels of worry and institutional trust (Prati, 2020). Trusted institutions can play an influential role in facilitating the widespread distribution and acceptance of the vaccine and greater trust in healthcare institutions reduces the risk, especially among the general population, of believing conspiracy theories about the virus’s origin and pharmaceutical companies’ views that the COVID-19 pandemic is a business opportunity (Loomba et al., 2021).

General vaccine-related attitudes have been found to predict coronavirus vaccine refusal and acceptance among Italian university students (Baccolini et al., 2021). In this study, vaccine hesitancy was predicted by being male, a non-healthcare student, at a lower academic level, and an undisclosed political position. On the other hand, greater perceptions of COVID-19 risks, concerns about the pandemic, beliefs in vaccine safety and effectiveness, and adherence to other preventive regimens were negatively associated with vaccine hesitancy. A longitudinal study conducted in the UK found that vaccine hesitancy was higher in women, younger people, the less educated, and ethnic subgroups (Robertson et al., 2021). The findings of these studies suggest that vaccine hesitancy is related to demographic variables (likely as markers for other psychological variables) and, more specifically, negative attitudes toward vaccines. Thus, clear information about the specific attitudes that are predictive of vaccine-hesitancy is needed to enable public health organisations and governmental institutions to maximise uptake of the COVID-19 vaccine.

As far as we know, there are no instruments validated in Italian designed to examine attitudes towards vaccines, which is particularly important in this time of the COVID-19 pandemic. However, Martin and Petrie (2017) developed the Vaccination Attitudes Examination (VAX) Scale, a short and comprehensive tool for identifying people with vaccination reluctance. It allows for the assessment of four different anti-vaccination attitude dimensions, consistent with the premise that more general attitudes can be predicted based on more specific ones (Littlejohn, 2002): (i) mistrust of vaccine benefit (i.e., people’s mistrust of a vaccine’s ability to protect against infectious diseases); (ii) worries over unforeseen future effects (i.e., people’s concern about potential side effects of vaccines); (iii) concerns about commercial profiteering (i.e., people’s wariness about the influence of the powerful pharmaceutical companies in the development and deployment of vaccines); and (iv) preference for natural immunity (i.e., the mistaken belief that natural immunity is superior to vaccinations) (Huynh & Senger, 2021; Martin & Petrie, 2017). These factors cover a wide range of reasons for vaccination hesitancy and refusal and based on a review of the literature and focus group data, capture the main dimensions on which such reluctance and refusal are typically based (Martin & Petrie, 2017).

Although these four dimensions are correlated, they are nonetheless distinct (Martin & Petrie, 2017), and prior literature shows that people’s anti-vaccination attitudes are not uniform and are related to demographic and other factors. For example, Godasi et al. (2021) demonstrated that people with lower education, low job skills, and joint families were more inclined to believe that vaccines have been marketed for commercial profiteering; and being married and living in a close family predicted preference for natural immunity. Moreover, of the various predictors, mistrust of vaccine benefits and concerns about future unforeseen effects were the most important for uncertainty and unwillingness to vaccinate against COVID-19 (Paul et al., 2021).

Recently, the VAX scale has been successfully validated in other languages, such as Romanian (Huza, 2020), Hebrew (Shacham et al., 2021), Spanish (Paredes et al., 2021), Turkish (Yildiz et al., 2021), and Telugu (Godasi et al., 2021). In addition, the VAX scale has also been adapted to specifically assess attitudes towards vaccines against COVID-19 (Shacham et al., 2021; Taylor et al., 2020).

Based on the reviewed literature, the present study aimed to examine the psychometric properties of the VAX scale (Martin & Petrie, 2017) in a general population in Italy. Specifically, the present study aimed to: 1) propose an Italian translation of the VAX scale (VAX-I); 2) examine the VAX-I scale’s psychometric properties and provide additional psychometric evidence of the VAX-I scale’s measurement invariance across gender (male vs female) since this was not assessed in the previous validation studies; and finally, 3) to assess the construct validity of the VAX-I scale.

Measurement invariance involves whether scores from the VAX-I scale have the same meaning across different conditions/groups or whether differences are created by diverse interpretations of the items in the instrument. Since the VAX scale was developed recently, no evidence of measurement invariance has been reported. However, this approach has been applied in a recent study evaluating the invariance of the DrVac-COVID19S across two cultural groups (Yeh et al., 2021). Testing measurement invariance is an essential feature of verifying the stability of scores in the VAX-I scale across gender groups, allowing healthcare and policymakers to use any differences in findings across subsamples (e.g., males vs females) to more effectively tailor COVID-19 vaccination messaging and campaigns (Shen, et al., 2021). Additionally, this information will be essential, in future research, for determining whether temporal variations in the VAX-I scale are the result of actual increases or decreases in the construct or merely artefacts of measurement (Yeh et al., 2021; Adamczyk et al., 2021).

Consequently, we hypothesised that the Italian VAX-I scale would confirm the original factorial structure (H1), would meet the criteria of the measurement invariance across gender (males vs females; H2), and would show a significant negative association with intention to get flu vaccine, health perceptions, and trust in health authorities (H3).

## Method

### Participants

The sample included 246 men (46%) and 288 women (54%). The participants’ ages ranged from 18 to 87 (*M* = 32.41, *SD* = 15.35). The educational level of the participants was quite high, with 41.57% of the participants having at least a college degree (bachelor/master/doctorate or comparable degree), and the others having a general qualification (elementary school/secondary school). Most of the participants were university students (48.3%), followed by employees (30.5%), and self-employed (11%).

### Measures

The Vaccination Attitudes Examination (VAX) Scale (Martin & Petrie, 2017) is a tool comprised of 12 items, each rated on a 6-point Likert-type scale (1 = *strongly disagree* to 6 = *strongly agree)*. Three of the items are reverse-coded. The composite scale may be further divided into four subscales: 1) mistrust of vaccine benefit (e.g., *“I feel safe after being vaccinated”*); 2) worries about unforeseen future effects (e.g., *“I worry about the unknown effects of vaccines in the future”*); 3) concerns about commercial profiteering (e.g., “*Authorities promote vaccination for financial gain, not for people’s health”*); and 4) preference for natural immunity (e.g., “*Natural immunity lasts longer than a vaccination”*), each indicated by three items. The Italian VAX-I scale displayed a good level of internal consistency (α = 0.94; ω = 0.94). Higher scores on the VAX-I represent more negative attitudes toward vaccinations (see Table S1).

Trust in health authorities (TAuth) was measured using three items adapted from Caso and colleagues (Caso et al., 2019) (e.g., *The COVID-19 vaccination program is safe because the Italian Health Ministry approves it*). Each item was measured using a 5-point Likert-type scale (1 = *strongly disagree* to 5 = *strongly agree*). Items were averaged to generate scores for trust in health authorities (α = 0.93; ω = 0.93).

Furthermore, three additional single items were administered to assess the construct validity of the VAX-I scale.

The first item was drawn from the Medical Outcomes Study’s General Health Perceptions scale (GHP; Stewart et al., 1992) and reads, “*In general, would you say your health is poor, fair, good, very good, or excellent*?” with a 5-point Likert-type response scale (1 = *poor* to 5 = *excellent*). This item has been used in many research studies, including nationally representative assessments of Americans from various ethnicities and income levels (Barger et al., 2006). It is considered a good indicator of self-reported health.

The second was a single item from the Perceived Sensitivity to Medicines scale (PSM; Horne et al., 2013). This item reads, “*My body is very sensitive to medicines*”, and responses are on a 5-point Likert-type scale (1 = *strongly disagree* to 5 = *strongly agree*).

The third query was regarding behavioural intentions, “*I intend to get the flu vaccine*” (﻿ItoFV), with a dichotomous (yes/no) response option.

Demographic information. Respondents were asked to indicate their age, the gender, the educational level, and the job.

### Procedure

An online survey comprised of demographic items and the study measures was created using the free software Google Forms®. The online survey was distributed from 17 September to 31 October 2021 across the national territory through social networking sites (e.g., Facebook, WhatsApp, and Instagram) together with a snowball recruiting technique. All the Italian participants were aware that participation in the study was voluntary, that all data would be collected anonymously, that data would only be used for scientific purposes, and all consented to participate under these terms. The online survey took approximately 10 min to complete. Approval for this study was obtained from the Ethical Committee of the Calabria Region (Catanzaro, Italy).

### Translation of the VAX-I Scale

A cross-cultural adaptation translation was conducted to assure equivalence between the original and Italian versions of the VAX scale. The translation process was performed by following the APA guidelines. First, a bilingual person independently translated items from the source language to the target language, then a different bilingual person then independently translated the items back into the source language. Last, a researcher compared the original with the back-translated version to establish if anything important was changed in the translation process. The result of the back translation confirmed the equivalence of the original and translated versions of the measures.

### Data Analyses

First, we computed descriptive statistics. Asymmetry and kurtosis were calculated for each item. The multivariate normality of the data was assessed by computing Mardia’s (1970) index (K). The Mardia’s skewness for the current data was, *K* = 28.81, *p* < 0.001, and the Mardia’s kurtosis was, *K* = 56.45, *p* < 0.001, indicating a deviation from multivariate normality. The violation of multivariate normality suggests the use of robust estimators. No missing item-level data were detected since answers were mandatory for all questionnaires. Finally, no other issues were detected after screening the data; therefore, no participants were excluded from the analyses.

Second, we explored the underlying structure of the VAX-I scale by conducting a parallel factor analysis, with principal axis factoring as the extraction method and an oblimin rotation, which assumes a correlation among factors. Finally, sampling adequacy and suitability of the data were evaluated with Kaiser–Meyer–Olkin (KMO) and Bartlett’s test of sphericity.

Third, the factor structure derived from the parallel factor analysis was verified using a confirmatory factor analysis (CFA) with maximum likelihood (MLM) parameter estimates with standard errors and a mean-adjusted chi-square test. This estimation method is also referred to as the Satorra-Bentler chi-square test (MLM χ^2^ S-B), which has been demonstrated to be robust with non-normal data (Satorra & Bentler, 1994). Following the recommendation of Hu and Bentler (1999), multiple indices were used to evaluate the model fit (adopted cut-offs in parentheses): the chi-square (χ^2^) test with associated p-value (*p* > 0.05), comparative fit index (CFI ≥ 0.95), Tucker–Lewis Index (TLI ≥ 0.95), root-mean-squared error of approximation (RMSEA ≤ 0.06) with 90% confidence interval, and standardised root mean square residual (SRMR < 0.08).

Fourth, an analysis of the internal reliability of the VAX-I scale was performed using two different indexes, Cronbach’s alpha (α) and McDonald’s omega (ω), respectively, as well as item-total correlations.

Fifth, multigroup confirmatory factor analysis (MGCFA) was conducted with MLM χ^2^ S-B to test for measurement invariance across gender groups (males vs females) with three nested models with increasing constraints (Kline, 2016): (1) configural invariance, the least restrictive model, in which all factor loadings and item intercepts are freely estimated for each group; (2) metric invariance, which assumes configural invariance and requires equality of the unstandardised pattern coefficients; and (3) scalar invariance, which assumes weak invariance and requires equal unstandardised intercepts across groups to confirm that different groups use the indicator’s response scale in the same way. All the models were statistically compared using the difference between the chi-square statistics with degrees of freedom equal to the difference in degrees of freedom between models. The ΔCFI value was used to test the between-group invariance of CFA models. According to Cheung and Rensvold (2002), invariance can be assumed when the absolute value is 0.01 or less.

Finally, correlational analyses and four independent samples *t*-tests were conducted to assess the scale’s validity in terms of other theoretically related constructs. Gender differences were also investigated.

Statistical analyses were conducted with SPSS Version 27 (IBM Corp., 2020), Mplus 7.2 (Muthén & Muthén, 2014) for the confirmatory factor analysis, and R’s *psych* package (Revelle, 2017) for the parallel factor analysis.

## Results

### Preliminary Analysis

The descriptive statistics for the VAX-I scale are reported in Table 1. The mean values of the VAX-I items ranged from 1.93 (Item_9) to 3.47 (Item_4).

**Table 1:** Descriptive statistics and item-total correlations for the VAX-I scale

Item	Min	Max	Mean	*SD*	Skewness	Kurtosis	Item-total correlation
Item_1r	1	6	2.49	1.40	0.86	0.02	0.62
Item_2r	1	6	2.11	1.31	1.23	0.90	0.56
Item_3r	1	6	2.39	1.36	0.96	0.27	0.67
Item_4	1	6	3.47	1.52	0.01	-1.06	0.60
Item_5	1	6	3.07	1.49	0.37	-0.85	0.70
Item_6	1	6	2.99	1.61	0.42	-0.96	0.75
Item_7	1	6	2.34	1.53	0.96	-0.20	0.80
Item_8	1	6	2.11	1.46	1.17	0.26	0.80
Item_9	1	6	1.93	1.36	1.48	1.30	0.80
Item_10	1	6	2.49	1.47	0.79	-0.38	0.78
Item_11	1	6	2.31	1.40	0.88	-0.19	0.75
Item_12	1	6	2.27	1.45	0.93	-0.12	0.76

*Note*. r = reverse scored

The corrected item-total correlation estimates (see Table 1) were higher than 0.40, supporting the internal reliability of the VAX-I scale (Nunnally & Bernstein, 1994).

### Parallel Analysis, Confirmatory Factor Analysis, and Invariance Tests

The results of the parallel factor analysis (see Table 2) confirmed the four-factor structure of the Italian VAX-I scale by showing an excellent fit to the data, χ^2^ (24, *N* = 534) = 41.08, *p* = 0.01, TLI = 0.99, RMSEA = 0.04, 90% CI = (0.02, 0.06), SRMR = 0.01, with 78.2% of the cumulative variance explained. The Kaiser–Meyer–Olkin (KMO) test of sampling adequacy was 0.91, indicating that the current data were suitable for subsequent analyses. Similarly, Bartlett’s test of sphericity was also good, χ^2^ (66, *N* = 534) = 5571.32, *p* < 0.001), indicating the appropriateness of the data for factor analysis.

**Table 2:** Factor loadings, the variance not explained for each item, and reliability

Item	F1	F2	F3	F4	Uniqueness
Item_1	0.86				0.25
Item_2	0.74				0.41
Item_3	0.98				0.06
Item_4		0.92			0.26
Item_5		0.74			0.29
Item_6		0.75			0.23
Item_7			0.73		0.22
Item_8			0.98		0.11
Item_9			0.79		0.18
Item_10				0.69	0.24
Item_11				0.93	0.16
Item_12				0.89	0.19
Cronbach alpha (α)	0.90	0.89	0.93	0.92	
McDonald’s omega (ω)	0.90	0.89	0.93	0.92	

*Note*. F1 = mistrust of vaccine benefit; F2 = worries about unforeseen future effects; F3 = concerns about commercial profiteering; F4 = preference for natural immunity

**Fig. 1:**
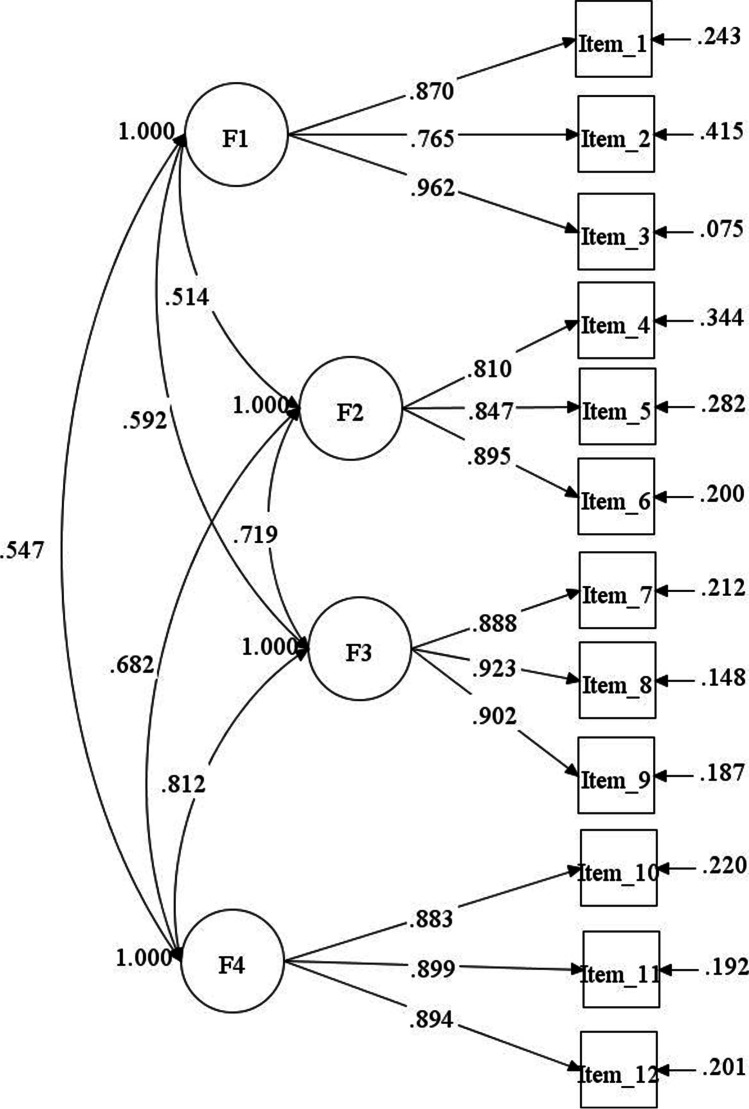
CFA results: Path diagram and standardised estimated parameters for the Italian VAX-I scale. Note. F1 = mistrust of vaccine benefit; F2 = worries about unforeseen future effects; F3 = concerns about commercial profiteering; F4 = preference for natural immunity. All factor loadings were statistically significant, *p* < 0.001

Confirmatory factor analysis (CFA) was next performed to test the factorial structure obtained from the parallel factor analysis. Since the data violated the assumption of normality, the MLM χ^2^ S-B estimator was used. The original four-dimensional factorial structure showed a good fit to the data, χ^2^ (48, *N* = 534) = 109.72, *p* < 0.001, CFI = 0.98, TLI = 0.98, RMSEA = 0.05, 90% CI = (0.04, 0.06), SRMR = 0.03. Figure 1 shows the results of the CFA with standardised factor loadings. Cronbach’s alpha and McDonald’s omega indicated a high degree of internal consistency for the scale and subscales (see Table 2). Additionally, the values of reliability did not change significantly when one item was deleted, indicating good internal consistency. These results suggested that the 12-item VAX-I is a reliable instrument to measure vaccine attitudes in the Italian context.

Measurement invariance for gender was tested by estimating the VAX-I models separately for males and females. The fit indices showed no differences between males and females (Table 3). This finding suggested that both genders had the same basic conceptualization of VAX-I and interpreted the items of each factor analogously.

**Table 3:** Test of the invariance of the Italian VAX-I scale across gender

	χ^2^	*df*	∆χ^2^	∆*df*	CFI	TLI	RMSEA	SRMR	ΔCFI
Male	81.19	48	-	-	0.983	0.977	0.053	0.035	-
Female	93.28	48	-	-	0.979	0.971	0.057	0.036	-
Configural	175.07	96	-	-	0.981	0.973	0.056	0.035	-
Metric	183.81	104	8.09^(ns)^	8	0.980	0.975	0.054	0.039	-0.001
Scalar	195.33	112	10.74^(ns)^	8	0.980	0.976	0.053	0.039	0.000

### Validity of the Construct

The results of the bivariate Pearson’s correlations (Table 4) showed a significant positive association between factor 2 of the VAX-I and the item assessing perceived sensitivity to medicines. Additionally, as predicted, a significant negative correlation was found between the four factors of the VAX-I scale, general health perceptions (GHP), intention to get the flu vaccine (ItoFV), and trust in health authorities (TAuth); and a significant, but weak, positive correlation was found between the VAX-I scale and perceived sensitivity to medicines.

**Table 4:** Pearson’s correlation, means, standard deviation (SD) for VAX-I subscales, and total score

	Mean	*SD*	1	2	3	4	5	6	7	8	9	10	11	12
1. Age	32.41	15.35	1.00											
2. Edu	3.59	1.05	0.38^***^	1.00										
3. Job	5.05	1.31	-0.53^***^	- 0.15^***^	1.00									
4. PSM	2.54	1.12	0.15^**^	0.04	-0.05	1.00								
5. GHP	3.37	0.87	-0.37^***^	-0.13^**^	0.23^***^	-0.21^***^	1.00							
6. ItoV	1.37	0.48	-0.02	-0.09^*^	0.10^*^	0.02	0.00	1.00						
7. TAuth	3.92	0.98	-0.05	-0.02	0.11^*^	-0.01	0.11^**^	0.16^***^	1.00					
8. F1	2.33	1.24	0.07	-0.02	-0.07	0.06	-0.20^***^	-0.17^***^	-0.67^***^	1.00				
9. F2	3.18	1.39	0.11^*^	-0.00	-0.12^**^	0.08^*^	-0.19^***^	-0.12^**^	-0.49^***^	0.45^***^	1.00			
10. F3	2.13	1.36	0.14^**^	-0.01	-0.14^**^	0.06	-0.16^***^	-0.10^**^	-0.55^***^	0.56^***^	0.65^***^	1.00		
11. F4	2.35	1.34	0.14^**^	-0.01	-0.14^**^	0.07	-0.14^**^	-0.09^*^	-0.47^***^	0.51^***^	0.61^***^	0.76^***^	1.00	
12. VAX-I	2.50	1.11	0.14^**^	-0.01	-0.14^**^	0.08^*^	-0.20^***^	-0.15^**^	-0.65^***^	0.74^***^	0.82^***^	0.89^***^	0.87^***^	1.00

*Note*. Edu = Education (from 1 = elementary school to 6 = master); Job (from 1 = unemployed to 6 = student); PSM = Perceived Sensitivity to Medicines Scale; GHP = General Health Perceptions Scale; ItoV = Intention to vaccination (1 = no, 2 = yes); TAuth = Trust in health authorities; F1 = mistrust of vaccine benefit; F2 = worries over unforeseen future effects; F3 = concerns about commercial profiteering; F4 = preference for natural immunity

^*^p < 0.05. **p < 0.01. ***p < 0.001

Independent-samples *t*-tests were conducted comparing VAX-I scale means for those who intended to get a flu vaccine and those who did not. As expected, individuals who did not intend to receive an influenza vaccination scored higher on all VAX-I dimensions than those who planned to be vaccinated (Table 5). The results of a *t*-test comparing genders indicated that females (*M* = 2.74, *SD* = 1.11) had significantly higher total VAX-I scores compared to males (*M* = 2.22, *SD* = 1.83), *t*(532) = -5.54, *p* < 0.001.

**Table 5:** Independent Samples t-test results of the difference among the total score of the VAX-I and intention to get the flu vaccine

	No (*N* = 336)		Yes (*N* = 198)					
	*M*	*SD*	*M*	*SD*	*t-*test	*df*	*p*	Effect size (Cohen’s *d*)
F1	2.49	1.25	2.06	1.17	3.89	532	0.001	0.35
F2	3.31	1.37	2.95	1.41	2.87	532	0.004	0.26
F3	2.24	1.36	1.94	1.34	2.53	532	0.012	0.23
F4	2.45	1.38	2.19	1.27	2.18	532	0.030	0.19

*Note*. F1 = mistrust of vaccine benefit; F2 = worries about unforeseen future effects; F3 = concerns about commercial profiteering; F4 = preference for natural immunity

## Discussion

Vaccination represents the most outstanding achievement of modern medicine and provides hope for preventing the spread of communicable diseases, including the ongoing COVID-19 pandemic. Unfortunately, worldwide anti-vaccination movements question the utility and safety of the COVID-19 vaccine and hesitancy about this vaccine is increasing (Shacham et al., 2021; Trujillo & Motta, 2021). Therefore, it is especially crucial now to understand the reasons for vaccine hesitancy and to identify the most suitable strategies for countering misinformation about vaccines. Considering this, the present paper adapted a measurement tool (VAX-I) to the Italian context, to better assess which factors predict anti-vaccination attitudes.

The results of the parallel factor analysis indicated that the Italian VAX-I scale has four distinct, but correlated factors confirming the original version of the VAX scale: (F1) mistrust of vaccine benefit, (F2) worries about unforeseen future effects, (F3) concerns about commercial profiteering, and (F4) preference for natural immunity. Furthermore, in line with the original study and the findings reported in previous translation and validation studies (Huza, 2020; Martin & Petrie, 2017; Yildiz et al., 2021), the confirmatory factor analysis indicated that the four-factor model provided the best fit to the current data (H1 was supported). In the present study, the results of the item-total correlation, Cronbach’s alpha, and McDonald’s omega coefficients indicated excellent internal consistency reliability of the VAX-I scale.

When testing the factorial structure in a multigroup analysis across gender, all three levels of constraint supported the invariant nature of the factor structure. As hypothesised (H2), the VAX-I scale functions equivalently for men and women. No previous studies have tested the gender invariance of the VAX scale, and this result provides a foundation for future investigations that measure gender differences in self-reported vaccine hesitancy.

Although the VAX-I demonstrated measurement invariance, findings indicated substantive differences in attitudes toward vaccinations for men versus women, with women reporting somewhat more negative attitudes than men. However, the actual results regarding gender differences are thus far equivocal. For example, in a prior study conducted in Italy among university students, males demonstrated greater vaccination hesitancy (Baccolini et al., 2021), whereas, in the UK, females scored higher (Robertson et al., 2021). Nevertheless, the current findings add another data point to the growing body of literature; future studies should endeavour to identify potential moderators of observed gender differences.

The VAX-I also demonstrated satisfactory construct validity (H3 supported). The Italian VAX-I scale exhibited a negative and significant correlation with the general perception of health (GHP), and intention to get the flu vaccination– consistent with previous studies (Huza, 2020; Martin & Petrie, 2017), and trust in health authorities. Those with higher VAX-I scores also reported being more sensitive to medicines in general. Consistent with a previous work, our results showed that those with lower trust in health authorities are more concerned about vaccine safety, suggesting that transparent communication about vaccines is urgent (Loomba et al., 2021).

In terms of demographic associations, correlation analyses indicated that older and unemployed Italians had more negative views about vaccines, but no associations were found for VAX-I scores and education. These findings suggest groups that may benefit from targeted messaging and information-sharing about vaccinations, particularly regarding safety and benefit to average people (vs corporations). The fact that the first factor (mistrust of vaccine benefit) was not correlated with age or occupational status indicates that individuals likely recognise that there is a benefit to vaccines, but the significant correlations with the other factors indicate that concerns about safety, profiteering and benefit in contrast with natural exposure are more effective.

Finally, the independent samples *t*-test indicated that people who were not planning to get the seasonal flu vaccine scored higher on each factor of the VAX-I scale compared to those who did plan to be vaccinated, which is consistent with findings of previous studies (Martin & Petrie, 2017; Taylor et al., 2020). From a public health perspective, the relative strength of associations between the four factors of the VAX-I and outcomes (intentions, behaviours) provide helpful information for designing intervention programs and public health campaigns that focus strategically on the most problematic areas. Based on the current results, Italian men and women score highest on the second factor of the VAX-I scale (worries about unforeseen future effects), suggesting that messaging focused primarily on safety might be most effective in countering vaccine hesitancy.

Therefore, the contribution of the present study is threefold. First, to our knowledge, this study is the first to adapt and validate an Italian version of the VAX scale. The VAX-I complements those already available in other languages (e.g., English, Turkish, Romanian, Hebrew, Spanish, Telugu), facilitating future comparative research on vaccine hesitancy. Second, the present study adopts more rigorous statistical methods and approaches, confirming the psychometric properties of the original version of the VAX scale. Third, the current findings provide some limited additional information regarding vaccine hesitancy in Italy and provide preliminary direction about which aspects of vaccination attitudes might be most effectively targeted for change in the future. Overall, the current findings suggest that the VAX-I scale is psychometrically sound for assessing vaccine hesitancy in the Italian context.

## Limitations

The present study has several limitations. First, only internal consistency reliability was assessed—test–retest reliability was not evaluated. Future research should confirm consistency over time and might also look at changes over time as a demonstration of the effectiveness of interventions aimed at changing attitudes. Another critical limitation is the exclusive use of self-report questionnaires with their potential biases (e.g., social desirability). Therefore, new studies should combine explicit and implicit measures to verify the accuracy of vaccination attitude reporting.

Since the four dimensions of the VAX-I scale focus on the cognitive and not the affective component of attitudes, future studies might extend the present scale by considering more affective aspects of resistance to vaccination (Crites et al., 1994) and examining the relationship between need for cognition (Cacioppo & Petty, 1982), need for affect (Maio & Esses, 2001) and scores on the VAX-I scale. Finally, given the well-known matching effect in persuasion (Aquino et al., 2020; Teeny et al., 2021) and the cognitive nature of the VAX-I dimensions, we might expect that more cognitively oriented people would receive more extreme scores on the VAX-I Scale. It would be interesting to examine this last possibility in future research.

## Theoretical and Practical Contribution

The good psychometric qualities of the Italian VAX-I scale suggest that it is a valid and reliable instrument for screening and research purposes, useful for health care providers in assessing attitudes toward vaccines and scholars interested in investigating anti-vaccination attitudes and their associations with other variables. In particular, based on scores obtained in the four different dimensions of the VAX-I scale, it is also possible to identify the predominant type(s) of anti-vaccination attitudes and, therefore, implement targeted intervention programs to promote their modification among the general population.

## Conclusion

The present study described the psychometric properties of the Italian VAX-I scale. The results of the parallel and confirmatory factor analyses showed that the four-factor structure of the VAX-I scale fits the data well, as in the original version. The measurement invariance analyses revealed that the VAX-I scale is stable across gender. Construct validity was supported by the significant negative correlation with general health perceptions, intentions to get the flu vaccine, and trust in health authorities, and the positive association with perceived sensitivity to medications. Therefore, the good psychometric qualities of the VAX-I scale suggest its utility for detecting vaccine-hesitancy in Italian people. Finally, using VAX-I subscale scores, it is also possible to understand the more nuanced nature of those attitudes against vaccines.

## Supplementary Information

Below is the link to the electronic supplementary material.

Supplementary file1 (PDF 87 KB)

## Data Availability

All data and materials are available from the first author upon request.
