# Supplementary material for: Validation and psychometric properties of the Italian Vaccination Attitudes Examination (VAX-I) scale
Source: Curr Psychol. 2022 May 27:1–11. Online ahead of print. doi: 10.1007/s12144-022-03209-5 (PMC9136196; doi:10.1007/s12144-022-03209-5)
Supplement: Supplementary file 1 — Supplementary file1 (PDF 87 KB) [file 12144_2022_3209_MOESM1_ESM.pdf]

The vaccination Attitudes Examination Scale (VAX-I)

Versione Italiana

Istruzioni. Queste domande sono progettate per aiutarci a comprendere meglio le convinzioni delle persone sulle vaccinazioni. Le chiedo di contrassegnare le risposte che riflettono più accuratamente i suoi sentimenti o le sue convinzioni. Non ci sono risposte giuste o sbagliate.

1. Mi sento al sicuro dopo essere stata/o vaccinata/o. (-)	Fortemente in Disaccordo 1 2 3 4 5 6 Fortemente D’accordo
2. Posso contare sui vaccini per fermare le malattie infettive gravi. (-)	Fortemente in Disaccordo 1 2 3 4 5 6 Fortemente D’accordo
3. Mi sento protetto/a dopo essermi vaccinato/a. (-)	Fortemente in Disaccordo 1 2 3 4 5 6 Fortemente D’accordo
4. Sebbene la maggior parte dei vaccini sembri sicura, potrebbero esserci dei problemi non ancora scoperti.	Fortemente in Disaccordo 1 2 3 4 5 6 Fortemente D’accordo
5. I vaccini possono causare problemi imprevedibili nei bambini.	Fortemente in Disaccordo 1 2 3 4 5 6 Fortemente D’accordo
6. Mi preoccupano gli effetti sconosciuti che i vaccini potrebbero avere in futuro.	Fortemente in Disaccordo 1 2 3 4 5 6 Fortemente D’accordo
7. I vaccini fanno guadagnare molti soldi alle aziende farmaceutiche, ma in realtà non sono utili alle persone.	Fortemente in Disaccordo 1 2 3 4 5 6 Fortemente D’accordo
8. Le autorità promuovono la vaccinazione a scopo di lucro e non per la salute delle persone.	Fortemente in Disaccordo 1 2 3 4 5 6 Fortemente D’accordo
9. I programmi di vaccinazione sono una grande truffa.	Fortemente in Disaccordo 1 2 3 4 5 6 Fortemente D’accordo
10. L’immunità naturale dura più a lungo di una vaccinazione.	Fortemente in Disaccordo 1 2 3 4 5 6 Fortemente D’accordo
11. L’esposizione naturale a virus e germi offre la protezione più sicura.	Fortemente in Disaccordo 1 2 3 4 5 6 Fortemente D’accordo
12. Per il sistema immunitario è più sicuro essere esposti in modo naturale alle malattie piuttosto che tramite la vaccinazione.	Fortemente in Disaccordo 1 2 3 4 5 6 Fortemente D’accordo

Calcolo dei punteggi. I punteggi della scala e delle sottoscale si ottengono dalle medie dei punteggi degli item (Item 1, 2, 3 = F1: sfiducia nei benefici dei vaccini; Item 4, 5, 6 = F2: preoccupazione per futuri effetti imprevisti; Item 7, 8, 9 = F3: preoccupazione per i profitti commerciali; Item 10, 11, 12 = F4: preferenza per l’immunità naturale). (-) Codifica inversa
